# Retraction to: A complex evolutionary relationship between HHV-6A and HHV-6B

**DOI:** 10.1093/ve/vez054

**Published:** 2019-11-23

**Authors:** Diego Forni, Rachele Cagliani, Mario Clerici, Uberto Pozzoli, Manuela Sironi

**Affiliations:** 1 Bioinformatics, Scientific Institute IRCCS E. Medea, Bosisio Parini 23842, Lecco, Italy; 2 Department of Physiopathology and Transplantation, University of Milan, Milan 20090, Italy; 3 IRCCS Fondazione Don Carlo Gnocchi, Milan 20148, Italy

The authors of “A complex evolutionary relationship between HHV-6A and HHV-6B” (doi: 10.1093/ve/vez043) have requested retraction of their paper. All authors agree with this action. Virus Evolution is retracting the paper because its findings are unreliable due to honest experimental error.

Specifically, analyses and conclusions were based on the retrieval of genome sequences from GenBank that were erroneous (accession numbers: KY239023.1, KY274487.1–KY274525.1, KY290171.1–KY290221.1, KY315520.1–KY315558.1). These genomes have been since corrected on GenBank (accession numbers: KY239023.2, KY274487.2–KY274525.2, KY290171.2–KY290221.2, KY315520.2–KY315558.2). Raw read data for the genome assemblies are available on SRA (accession numbers: SRR8717229, SRR8745772–SRR8745811, SRR8749118–SRR8749151, SRR8749387–SRR8749420, SRR8757190–SRR8757227, SRR9736967–SRR9737010).

Since this mistake affects a significant portion of the paper’s results the journal has decided that a retraction, rather than a correction, is the most appropriate course of action. Virus Evolution apologises for any inconvenience caused by this retraction, and thanks the authors for their rapid action and open communication in dealing with this matter.

